# Kindlins, Integrin Activation and the Regulation of Talin Recruitment to αIIbβ3

**DOI:** 10.1371/journal.pone.0034056

**Published:** 2012-03-23

**Authors:** Bryan N. Kahner, Hisashi Kato, Asoka Banno, Mark H. Ginsberg, Sanford J. Shattil, Feng Ye

**Affiliations:** Department of Medicine, University of California San Diego, La Jolla, California, United States of America; Kings College London, United Kingdom

## Abstract

Talins and kindlins bind to the integrin β3 cytoplasmic tail and both are required for effective activation of integrin αIIbβ3 and resulting high-affinity ligand binding in platelets. However, binding of the talin head domain alone to β3 is sufficient to activate purified integrin αIIbβ3 *in vitro*. Since talin is localized to the cytoplasm of unstimulated platelets, its re-localization to the plasma membrane and to the integrin is required for activation. Here we explored the mechanism whereby kindlins function as integrin co-activators. To test whether kindlins regulate talin recruitment to plasma membranes and to αIIbβ3, full-length talin and kindlin recruitment to β3 was studied using a reconstructed CHO cell model system that recapitulates agonist-induced αIIbβ3 activation. Over-expression of kindlin-2, the endogenous kindlin isoform in CHO cells, promoted PAR1-mediated and talin-dependent ligand binding. In contrast, shRNA knockdown of kindlin-2 inhibited ligand binding. However, depletion of kindlin-2 by shRNA did not affect talin recruitment to the plasma membrane, as assessed by sub-cellular fractionation, and neither over-expression of kindlins nor depletion of kindlin-2 affected talin interaction with αIIbβ3 in living cells, as monitored by bimolecular fluorescence complementation. Furthermore, talin failed to promote kindlin-2 association with αIIbβ3 in CHO cells. In addition, purified talin and kindlin-3, the kindlin isoform expressed in platelets, failed to promote each other's binding to the β3 cytoplasmic tail *in vitro*. Thus, kindlins do not promote initial talin recruitment to αIIbβ3, suggesting that they co-activate integrin through a mechanism independent of recruitment.

## Introduction

Integrins are heterodimeric, transmembrane αβ adhesion receptors responsible for cell-cell and cell-matrix interactions during embryonic development, responses to injury, and pathological processes including athero-thrombosis and neoplasia. In mammalian cells, the ligand-binding affinity of many integrins can be regulated by “inside-out” signals, leading to propagated conformational changes across the plasma membrane that increase ligand binding affinity [1]. Studies *in vitro* and *in vivo* indicate that this form of integrin activation requires the binding of talin to the cytoplasmic tail of the integrin β subunit [2]. In unstimulated platelets, for example, talin is localized primarily to the cytoplasm [3] where it is thought to be “auto-inhibited” by virtue of the masking of an integrin-binding interface within the FERM domain of the N-terminal talin head domain (THD) by a region of the C-terminal talin rod domain [4], [5], [6]. Inside-out signals are hypothesized to release talin auto-inhibition, enabling recruitment of the protein to the plasma membrane where it can then interact with integrin β tails. This results in reorganization of the α and β subunit cytoplasmic and transmembrane domains and integrin activation [2], [7], [8].

In platelets [9], [10] where αIIbβ3 is the most abundant integrin, and in a CHO cell model system used to study αIIbβ3 signaling [11], [12], some of the key intracellular signals involved in agonist-dependent talin recruitment to αIIbβ3 have been identified. These include activation of the Rap1 GTPase, formation of a membrane-associated Rap1-GTP/RIAM adapter complex, and interaction of RIAM with talin [13]. At the molecular level, αIIbβ3 activation requires a series of interactions of the THD with the β3 tail, including the strong interaction with membrane-distal β3 tail residues centered at ^744^NPLY^747^, and additional interactions with membrane-proximal β3 tail residues and plasma membrane phospholipids [14], [15], [16].

**Figure 1:**
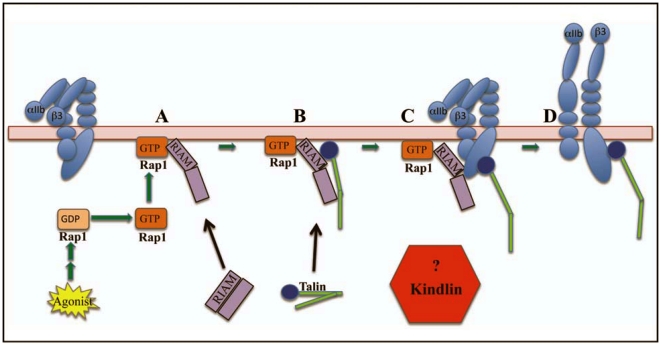
Model of agonist-induced αIIbβ3 activation. (**A**) Stimulation of a platelet agonist receptor (e.g., PAR1) by an agonist leads to the activation of Rap1, resulting in targeting of its effector, RIAM, to the plasma membrane. (**B**) Cell stimulation also releases talin from its auto-inhibitory state, resulting in separation of the THD from the talin rod domain and recruitment of talin to the membrane-bound Rap1/RIAM complex. (**C**) Membrane-bound talin is recruited to αIIbβ3 by interaction of the THD with membrane-distal residues in the β3 cytoplasmic domain. (**D**) Further interactions of the THD with membrane-proximal β3 tail residues and membrane phospholipids leads to separation of the αIIb and β3 tail and transmembrane domains, triggering propagated changes in the extracellular domains leading to high-affinity binding of adhesive ligands, such as fibrinogen. While kindlins, like talin, can interact with the β3 cytoplasmic tail, they can also bind to other proteins [20], [42], and the molecular basis of their integrin co-activating function remains unclear. This working model is based on published studies summarized in [2].

THD interaction with β3 is sufficient for αIIbβ3 activation when tested in the context of recombinant proteins and membrane lipid nanodiscs [17]. However, αIIbβ3 activation in platelets also requires kindlin-3, a hematopoietic cell-selective member of the kindlin family of adapter molecules [18], [19], which includes kindlin-1 and kindlin-2 [20], [21]. Although tissue distribution of the kindlins varies, all members of the family appear capable of engaging integrin β tails in a manner distinct from talin. For example, the interaction of αIIbβ3 with kindlin-3 or kindlin-2, which is normally expressed in CHO cells, requires β3 tail residues (^756^NITY^759^) that are membrane-distal to the talin-binding ^744^NPLY^747^ residues [20], [21], [22]. Importantly, kindlins alone appear to be less efficient than THD for αIIbβ3 activation [22], [23], [24]. Thus, the precise relationships between talin and kindlins during inside-out integrin signaling remain unclear. Furthermore, disruption of an agonist-induced signaling pathway leading to talin function can result in severe defects in inside-out integrin activation, as in the case of Rap1b deficiency in platelets [9]. Conceivably, kindlin could function at one or more loci of this signaling pathway (Figure 1).

Here we investigated whether kindlins influence talin recruitment to αIIbβ3, one of the hypotheses proposed to explain the mechanism of kindlin function [2], [20], [21], [25]. Using complementary approaches with intact cells and purified, recombinant proteins, we establish that kindlins do not promote talin recruitment to plasma membranes or to αIIbβ3. Conversely, talin does not promote the interaction of kindlins with αIIbβ3. These results indicate that kindlins might promote integrin activation by playing a role in events other than initial talin recruitment to integrin αIIbβ3.

## Methods

### Reagents and plasmid vectors

SFLLRN, an agonist peptide specific for human PAR1 [26] and antibody to the Flag epitope were from Sigma-Aldrich (St. Louis, MO). Antibodies specific for the external portion of the IL2 receptor (7G7B6, “Tac”), the human integrin β3 C-terminus (Rb 8275), αIIb (Rb2308), αIIbβ3 (D57) and activated αIIbβ3 (PAC-1) have been described [27], [28], [29]. Antibodies to β-actin, talin and calnexin were from Abcam (Cambridge, MA); antibody to the HA-epitope from Covance (Princeton, NJ); antibody to RhoGDI from Santa Cruz Biotechnology (Santa Cruz, CA); and antibodies to GFP and the His6 tag from Clontech (Mountain View, CA). Alexa Fluor-568, Alexa Fluor-647 and R-phycoerythrin-conjugated secondary reagents were from Invitrogen (Carlsbad, CA). Kindlin-2-specific antibody was a gift from Dr. Cary Wu, University of Pittsburgh, Pittsburgh, PA [24].

Plasmids encoding cDNAs for mouse talin1 [12], human PAR1 [30] and human kindlin-2 were sub-cloned into the pcDNA4/TO tetracycline-inducible expression vector (Invitrogen, Carlsbad, CA). Where indicated, GFP or DsRed (Clontech, Mountain View, CA) was used as a transfection marker.

### Cell culture and transfection

CHO-K1 [31], 293T [32] and NIH3T3 [33] cells were cultured in Dulbecco's Modification of Eagle's Medium (Cellgro, Manassas, VA) and supplemented with antibiotics, nonessential amino acids, L-glutamine and 10% fetal bovine serum. For transient transfections, Lipofectamine (Invitrogen, Carlsbad, CA) was used according to the manufacturer's recommendations. To produce stable cell lines, CHO cells were transfected with the appropriate expression plasmids. Forty-eight hours later, antibiotics for selection were added and cells were cultured for ∼2 weeks. Clones were selected further by single-cell sorting using MoFlo (Dako, Carpinteria, CA) and stable expression of recombinant proteins was confirmed by flow cytometry or western blotting. Stable CHO cell clones capable of tetracycline-inducible expression of PAR1, talin and kindlin-2 were generated as described [34].

### Kindlin-2 knockdown and integrin activation in CHO cells

The sequence of CHO cell *kindlin-2* was determined by reverse transcriptase PCR, and two shRNA sequences were designed as follows: shRNA1: 5′-AAAAAAGTGCACCGCCGGACTCCTCTCAGCTCTCACAAGCTTCTAAGAGCCGAGAGGAGTCCAGCGGTACACGTGTTTCGTCCTTTCCACAA-3′; shRNA2: 5′-AAAAAAGCTA ATCTGCAGACTACCATCCAGCGTACCAAGCTTCGCACACTGGATGGCAGCCTGCAGATTAGCGGGTTTCGTCCTTTCCACAA-3′. These shRNAs and a control shRNA obtained from a scrambled sequence of mRNA derived from Rock1 [35] were subcloned into lentiviral vector FG12 [36]. Depending on the experiment, GFP, DsRed or Tac was subcloned into FG12 downstream of the UbiC promoter to subsequently mark transduced cells. CHO cells were transduced with lentiviruses as described [35].

Integrin activation of transduced cells was assessed by flow cytometry with antibody PAC-1 [11]. Ninety-six hours after transduction, cells were incubated for 20 hours with 1 µg/ml doxycycline (or vehicle) to induce PAR1 and talin expression. Cells were then incubated for 20 min at room temperature with 100 µM SFLLRN (or vehicle), and specific (EDTA-inhibitable) PAC-1 binding to single living cells was quantified. To rescue the effect of the knockdown, cells transduced with *kindlin-2* shRNA were transiently co-transfected with kindlin-2 expression vector and a transfection marker for 52 hours. Cells were induced with doxycycline as indicated and specific PAC-1 binding to transduced, transfected cells was quantified.

### Protein interactions in living cells determined by bimolecular fluorescence complementation (BiFC)

Chimeric proteins αIIb-VC (VC: Venus C-terminal moiety) and VN-talin (VN: Venus N-terminal moiety) used for BiFC experiments have been described [11]. A VN-kindlin-2 chimera was produced by PCR, cloned into pCMV and sub-cloned into pcDNA4/TO for inducible expression in CHO cells [11]. Protein interactions involving VN-talin or VN-kindlin-2 and αIIb-VCβ3 were monitored by BiFC using flow cytometry [11] or deconvolution microscopy. As specified in each experiment, transfected or transduced cells were identified by expression of a fluorescent marker, such as DsRed, or Tac, the latter detected with a fluorescently-labeled secondary antibody [28]. In all experiments, BiFC signals were first normalized for αIIbβ3 expression detected with antibody D57 and compared across various experimental conditions.

For deconvolution microscopy, cells were plated onto fibrinogen-coated cover slips for 45 min and fixed by 4% paraformaldehyde in PBS for 10 min., stained with antibody D57 to αIIbβ3, and imaged with a deconvolution microscope (DeltaVision, Applied Precision, Issaquah, WA) using a charged-coupled device camera system (CoolSNAP HQ; Photometrics, Tucson, AZ) attached to an inverted wide-field fluorescence microscope (Eclipse TE200; Nikon, Melville, NY) equipped with 40× oil-immersion objective (Nikon, Melville, NY)). Identical software settings were used for image acquisition of all samples in a given experiment, and images were deconvoluted with an iterative-constrained algorithm. Cell fluorescence intensity range was standardized using the Softworks Analysis Program (Applied Precision, Issaquah, Washington). Any adjustments of color balance made with Photoshop CS4 software (Adobe Systems, San Jose, CA) were applied to the entire image in a figure, and no nonlinear adjustments were made. To examine cell spreading, images were assessed using ImageJ 1.41o software (National Institutes of Health, Bethesda, Maryland). Every cell in an image was selected. Cells that did not express the DsRed transduction marker or were not completely in the field-of-view were exempted and “holes” inside the cell were removed. Spreading was calculated as total pixels per cell. The mean pixel count for 50–75 cells for each treatment was acquired.

To evaluate talin and integrin co-localization by fluorescence overlay, transduced (DsRed-positive) cells were analyzed using Metamorph (Molecular Devices, Inc., Sunnyvale, CA). Cell fluorescence intensity range was standardized for each wavelength and the same threshold was used for each image. Cell membrane edges were selected by the “freehand” tool. Using “region statistics,” integrated intensities for the 525 nm (BiFC) and 684 nm wavelengths (αIIbβ3) were acquired for the membrane edge region, yielding total pixels for BiFC and αIIbβ3 (D57) fluorescence. A ratio describing the amount of BiFC fluorescence that overlaps the integrin fluorescence was defined as (BiFC integrated intensity)/(Integrin integrated intensity), and the mean ratio for 30–60 cells per treatment was calculated.

### Protein purification and β3 integrin tail pull-down assay

THD was expressed and purified [17]. Kindlin-3 was expressed and purified from S2 insect cells. Briefly, S2 insect cells stably expressing kindlin-3 with Flag and His6 purification tags were generated by selecting against blasticidin for two weeks. Cells were cultured, harvested and lysed in TBS buffer (20 mM Tris, 150 mM NaCl, pH 7.4), and kindlin-3 was purified sequentially with Ni-NTA, anion exchange and size exclusion columns (Figure S2).

β3 integrin tail pull-down assays were performed as described [37], [38]. Briefly, recombinant β3 tails were captured by Neutravidin beads (Thermo Fisher Scientific, Rockford, IL) and incubated at 4°C overnight with purified THD and kindlin-3 in a pull-down buffer (20 mM PIPES, 50 mM NaCl, 150 mM sucrose, 1 mM Na_3_VO_4_, 50 mM NaF, 40 mM Na_4_P_2_O_7_, 0.1% Triton X-100, pH 6.8). Beads were washed and the bound fraction was eluted with 1× sodium dodecyl sulfate-polyacrylamide gel electrophoresis (SDS-PAGE) loading buffer. Proteins bound to the β3 tail beads were separated by SDS-PAGE, transferred to a nitrocellulose membrane and detected by western blotting with anti-His6 antibody. Protein bands on blots were quantified with the Odyssey infrared imaging system (Li-Cor Biosciences, Lincoln, NE).

### Plasma membrane isolation

Cells were transfected with HA-talin and GFP-RIAM-(1-176)-CAAX [13] and subjected to surface biotinylation with 3 mM EZ-Link Sulfo-NHS-Biotin (Pierce Biotechnology, Inc., Rockford, IL) dissolved in 0.1 M sodium phosphate buffer, pH 8.0. After 30 min incubation, cells were extensively washed with phosphate-buffered saline, pH 7.4, and resuspended on ice for 10 min in fractionation buffer (20 mM HEPES-KOH pH 7.5, 1.5 mM MgCl_2_, 5 mM KCl, 0.2 mM Na_3_VO_4_, 10 µg/mL leupeptin, 10 µg/mL aprotinin, 1 mM phenylmethanesulfonylfluoride, and Complete mini protease inhibitor tablet (Roche Applied Bioscience, Indianapolis, IN)). Swollen cells were disrupted by passing through a 27G needle. The resulting mixture was centrifuged at 2000 rpm for 10 min to pellet nuclei and unbroken cells. The supernatant was further centrifuged at 14,000 rpm for 30 min to pellet the membrane fraction. The supernatant was saved as the “cytosolic” fraction.

Pelleted crude membranes were resuspended in fractionation buffer and incubated with BcMag™ Streptavidin Magnetic Beads (Bioclone Inc, San Diego, CA) for an hour at room temperature with rotation to obtain bound plasma membranes. After washing the beads, bound proteins were eluted with SDS sample buffer, separated on SDS-PAGE gels and transferred to a nitrocellulose membrane. The amounts of HA-talin and GFP-RIAM-(1-176)-CAAX in whole cell lysates, nuclear/intact cell, cytosolic, crude membrane, and plasma membrane fractions were analyzed by western blotting using anti-HA and anti-GFP antibodies, respectively. Antibodies to αIIb, calnexin, and RhoGDI were used as plasma membrane, ER membrane, and cytosolic markers, respectively. Bands corresponding to talin and αIIb in the whole cell lysates and in the plasma membrane fraction were scanned and quantified with the Odyssey imaging system. Talin detected in the plasma membrane fraction was normalized to the total amount in whole cell lysate and designated as “percent of total”. Since there might be variations in the efficiency of plasma membrane recovery for different cell preparations, the percent of total for talin was further normalized by the percent of total for αIIb, which as an integral membrane protein represents maximum possible plasma membrane recovery.

### Western blotting

Cells were lysed with NP-40 lysis buffer (50 mM Tris-HCl, pH 7.4, 1% NP-40, 150 mM NaCl, 1 mM NaF, 0.5 mM sodium vanadate, 12.5 µg/ml leupeptin, and Complete, EDTA free (Roche, Indianapolis, IN)). After clarification, lysates were subjected to SDS-PAGE, transferred to nitrocellulose membranes and analyzed by western blotting.

## Results and Discussion

αIIbβ3 activation in platelets is regulated by signaling pathways downstream of plasma membrane receptors, many of them G-protein-coupled [39]. Agonist peptide, SFLLRN [26], stimulates the human PAR1 thrombin receptor, a prototype agonist receptor . In order to study relationships between kindlins, talin and αIIbβ3, we turned to a CHO cell model system in which specific proteins can be silenced with shRNAs or expressed in a tetracycline-regulated manner, and αIIbβ3 activation can be monitored by flow cytometry using activation-specific antibody PAC-1. This model system exhibits talin-dependent αIIbβ3 activation in response to SFLLRN and over-expression of activated Rap1 and/or its effector, RIAM [11], [12]. Furthermore, over-expression of kindlin-2 enhances talin-induced αIIbβ3 activation in these CHO cells [21]. Thus, this CHO cell model has been employed to study inside-out integrin signaling through talin and RIAM with results consistent with those obtained from platelets and megakaryocytes [11].

**Figure 2:**
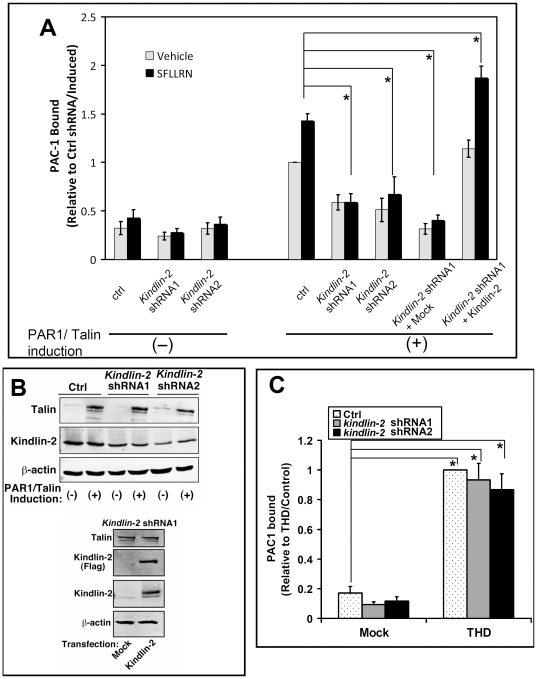
Kindlin-2 requirement for talin-dependent, agonist-induced αIIbβ3 activation in CHO cells. (**A**) Agonist-induced PAC-1 binding determined in kindlin-2 knockdown cells. αIIbβ3 CHO cells engineered to conditionally express PAR1 and talin were transduced with lentivirus encoding control (Ctrl) or *kindlin-2* shRNAs as described in Experimental Procedures. Cells were incubated for 20 min at room temperature with 100 µM SFLLRN (or vehicle), and specific PAC-1 binding was quantified by flow cytometry as described [11]. To control for off target effects of knock-down constructs, shRNA-transduced cells were transiently co-transfected with an shRNA-resistant form of Flag-kindlin-2 (or empty vector, Mock) and Tac as a transfection marker. After induction of PAR1 and talin with doxycycline, specific PAC-1 binding was measured and normalized to integrin expression as determined by D57 staining. For clarity, data are expressed as the fold-increase in PAC-1 binding relative to binding observed with doxycycline-induced cells transduced with control shRNA. Data represent means ± SEM of six independent experiments (asterisk, P<0.01). (**B**) western blots were performed to assess expression of talin and kindlin-2 in lysates of cells studied in panel A. β-actin was monitored as a loading control. In the kindlin-2 rescue experiments, kindlin-2 was assessed both with an antibody to kindlin-2 and an antibody to the Flag epitope. The cell lysates were from both uninfected and virus transduced cells whereas using flow cytometry gating, only virus transduced cells were analyzed in panel A. (**C**) Kindlin-2 shRNA has no effect on PAC-1 binding induced by THD. αIIbβ3 CHO cells were transduced with lentivirus encoding *kindlin*-2 (or control) shRNA. Cells were transfected as indicated with THD, empty vector (Mock) and DsRed. PAC-1 binding to transfected cells was quantified by flow cytometry. PAC1 binding was normalized to PAC1 binding when integrins are fully activated by an activating antibody, which also is sensitive to the integrin expression level [28]. For clarity, data are expressed as the fold-increase in PAC-1 binding relative to binding observed with THD transfected/control shRNA transduced cells. Data represent means ± SEM of 7 experiments. (Asterisk, P<0.01 against mock transfected/control shRNA transduced cells).

First, we designed *kindlin-2* shRNAs and performed preliminary experiments to characterize the *kindlin-2* lentiviral shRNA constructs. We observed greater than 90% knockdown of kindlin-2 in the shRNA-transduced cells (Figure S1). Next we asked whether endogenous kindlin-2 is required for SFLLRN-induced αIIbβ3 activation. CHO cells stably expressing αIIbβ3 and conditionally-expressing PAR1 and full-length talin in response to doxycycline [11] were transduced with lentiviruses expressing either a *kindlin-2* shRNA or a scrambled, control shRNA. PAC-1 binding to the transduced (GFP-positive) cells was analyzed by flow cytometry [11]. In the absence of doxycycline, cells transduced with control shRNA exhibited a relatively low level of PAC-1 binding, whether or not they had been stimulated with SFLLRN (Figure 2A). However, after induction of PAR1 and full-length talin with doxycycline, the cells exhibited higher basal PAC-1 binding, and significantly more PAC-1 binding in response to 100 µM SFLLRN (P<0.01) (Figure 2A). By contrast, cells transduced with either of two *kindlin-2* shRNAs and incubated with doxycycline showed less basal PAC-1 binding, and no enhanced PAC-1 binding in response to SFLLRN (Figure 2A). Thus, the silencing of endogenous *kindlin-2* abolishes agonist-induced αIIbβ3 activation. The inhibitory effect of kindlin-2 knockdown on αIIbβ3 activation could not be explained by off-target effects on expression levels of talin (Figure 2B) or PAR1 (not shown), and the specificity of kindlin-2 knockdown was supported by the observation that co-expression of a shRNA-resistant form of *kindlin-2* cDNA rescued the reduced integrin activation (Figure 2A,B). Thus, re-introducing kindlin-2 into the cell restores the increase in PAC-1 binding in response to SFLLRN (P<0.01). In contrast to its inhibitory effect on αIIbβ3 activation by talin, kindlin-2 knockdown had no effect on PAC-1 binding when recombinant THD was introduced into the CHO cells (Figure 2C). Previous work by others [21], which has been discussed in recent reviews [20], [40], showed a modest decrease in THD-induced integrin activation when kindlin-2 was depleted [21]. In contrast, we saw no statistically significant effect. Our results are consistent with previous findings that THD can activate αIIbβ3 mutants with markedly reduced affinity for kindlins [17], [41]. Our results also agree with the observation that THD alone is sufficient to activate integrins reconstituted in lipid bilayers [17]. Importantly, the same degree of kindlin-2 depletion reduced integrin activation mediated by full length talin (Figure 2). Thus as for kindlin-3 in platelets [18], kindlin-2 in CHO cells is required for αIIbβ3 activation in response to PAR1-mediated, talin-dependent inside-out signaling. That said, loss of kindlin or kindlin-integrin interaction only has a modest effect at best on activation of αIIbβ3 by THD [17], [21], [41].

**Figure 3:**
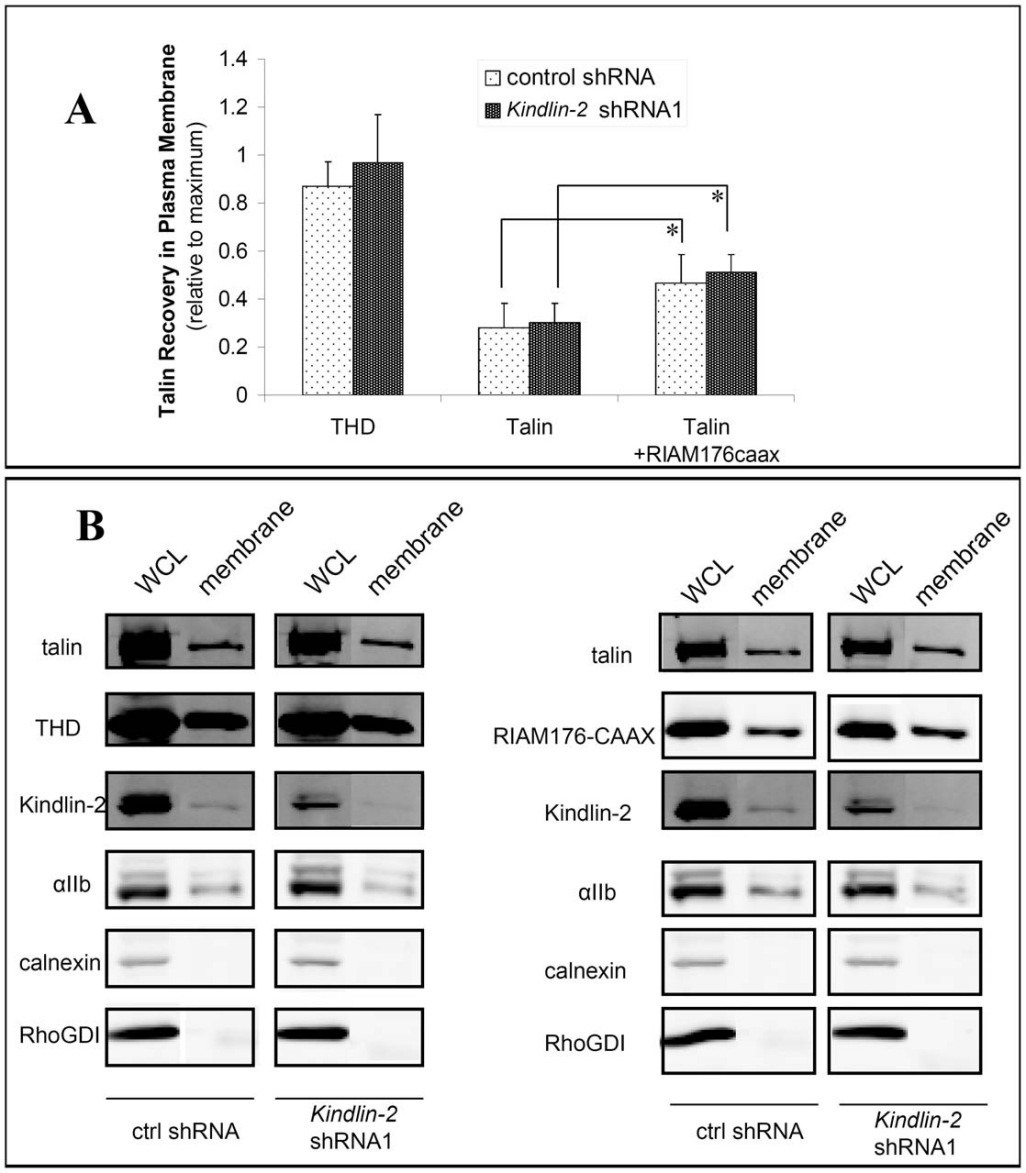
Effect of kindlin-2 knockdown on talin recruitment to the membrane. (**A**) αIIβb3 CHO cells were transduced with lentivirus encoding either control shRNA or *kindlin*-2 shRNA. Cells were then transfected as indicated with THD, talin, or talin and RIAM1-176CAAX. Intact cells were surface biotinylated in order to isolate membrane-bound proteins. Cells were broken-up by shear and then underwent serial centrifugation to isolate the nuclear/intact cell fraction, cytosolic fraction and crude membranes. The crude membranes were further purified with streptavidin conjugated beads. The streptavidin bound material was isolated as the plasma membrane fraction. The amount of THD and talin in each fraction as well as in whole cell lysate (WCL) was quantified by western blot. Data is expressed as relative protein recovery normalized to the recovery of integrin αIIb subunit in plasma membrane. Data represent means ± SEM of three independent experiments (asterisk, P<0.10 in paired t-test). (**B**) Representative western blots of the subcellular fractionation experiments showing the WCL and plasma membrane fraction. Western blot of the WCL showed that αIIb expression levels are unchanged. RhoGDI serve both as a loading control [46], [47] and a cytosolic marker. Western blots of each target proteins and markers were cut and juxtaposed for clarity. The complete blot images with all the subcellular fractions are shown in Figure S3.

These results could in principle be explained by a role for kindlin-2 in one or more steps of the inside-out αIIbβ3 signaling pathway illustrated in Figure 1. Kindlins contain a PH domain interposed between a split F2 subdomain within the integrin-binding FERM domain [42]. Since the PH domain may promote kindlin interactions with membrane phosphoinositides [43], we wondered if kindlins played a role in targeting of full-length talin to the plasma membrane. A role for the kindlin-2 PH domain in integrin function is supported by observations that deletion of this domain reduces β3 integrin adhesive function in CHO cells [21], whereas mutation of the PH domain abrogates kindlin-2 binding to phosphoinositides, particularly PIP_3_, and reduces β1 and β3 integrin activation and function in podocytes [44]. To determine whether kindlin-2 promotes talin recruitment to plasma membranes, αIIbβ3-CHO cells were surface-biotinylated, transduced with control or *kindlin-2* shRNA, and transfected with full-length talin and THD. Since cells transduced with *kindlin-2* shRNA2 grow slowly, we focused on shRNA1 transduced cells in order to consistently obtain sufficient number of cells for these experiments. Cells co-transfected with full-length talin and RIAM-(1-176)-CAAX, which targets talin to cell membranes [13], were used as positive controls for increased membrane recruitment. Cell plasma membranes were affinity-isolated using streptavidin, and the amount of associated talin was quantified by western blotting. Co-expression of αIIb and β3 subunits results in efficient surface expression of integrin αIIbβ3 [45]. Fluorescence images of αIIbβ3-expressing CHO cells in this work (see below) and others [12] verify that the majority of this integrin is at the cell surface. Furthermore, western blotting of whole cell lysates showed that αIIbβ3 expression levels were similar between the control cells and kindlin-2 knockdown cells when the loading control, endogenous RhoGDI [46], [47], was comparable (Figure 3B). Thus recovery of αIIb, an integral membrane protein, in the plasma membrane fraction was set as a reference for maximal plasma membrane recovery, and recovery of full-length talin or THD with plasma membranes was normalized to that of αIIb. RIAM-(1-176)-CAAX increased the abundance of full-length in plasma membranes (n = 3), although not to a level seen with THD. Kindlin-2 knockdown had no effect on the recovery of talin or THD within the plasma membrane fraction (Figure 3A,B). These results suggest that kindlin-2 is not required for the regulated targeting of talin to plasma membranes.

**Figure 4:**
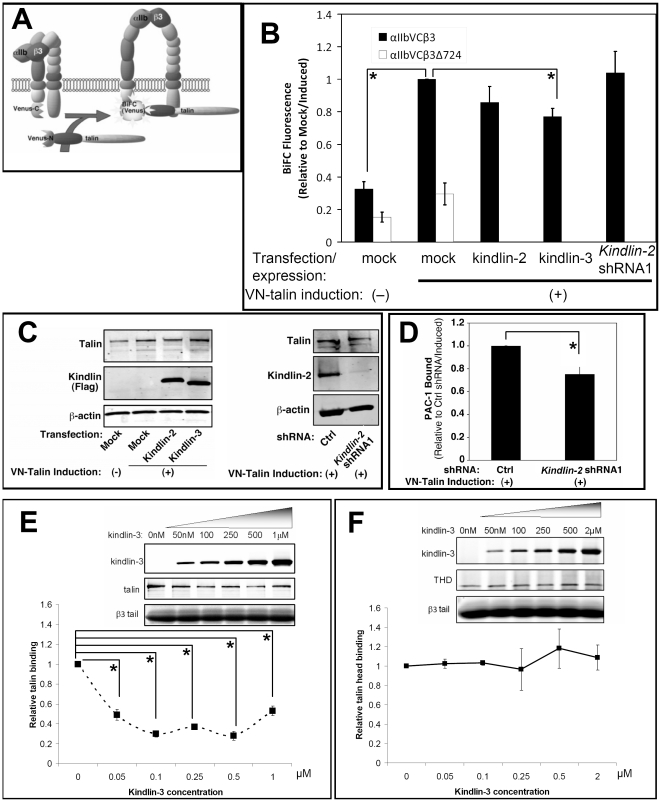
Effect of kindlin over-expression or depletion on talin interaction with αIIbβ3 in living cells and *in vitro*. (**A**) Schematic illustration of BiFC in CHO cells depicting αIIb-VC, VN-talin, and β3. When VN-talin interacts with αIIb-VCβ3 through the β3 tail, VN and VC should reconstitute the Venus fluorophore, resulting in BiFC [11]. (**B**) Neither over-expression nor knock-down of kindlins promote talin interaction with αIIbβ3. αIIb-VCβ3 CHO cells (or cells expressing mutant αIIb-VCβ3Δ724) were co-transfected with an expression vector for Flag-kindlin-2, Flag-kindlin-3, or empty vector (Mock) along with Tac as a transfection marker. To assess the effects of kindlin-2 knockdown, αIIb-VCβ3 CHO cells were transduced with *kindlin*-2 shRNA1 (or control shRNA, Mock). VN-talin expression was induced with doxycycline, and BiFC was quantified by flow cytometry. BiFC fluorescence was normalized to αIIbβ3 expression and presented as fold-increase relative to doxycycline-induced, mock-treated cells. Data represent means ± SEM of three experiments (asterisk denotes statistically significant difference against mock/induced, P<0.05). (**C**) Western blots were performed to monitor expression of talin and kindlins in lysates of αIIb-VCβ3 CHO cells studied in panel B. Left side: western blot of WCL showed successful expression of kindlin-2, kindlin-3 and VN-talin. Right side: Western blots of WCL showed successful knockdown of kindlin-2 by shRNA1, without effects on VN-talin expression. (**D**) Kindlin-2 knockdown decreased basal PAC-1 binding as expected (asterisk, P<0.01). (**E**) Purified talin with or without addition of kindlin-3 was incubated with the recombinant β3 cytoplasmic tails conjugated to neutravidin beads as described in Experimental Procedures. After washing, proteins bound to the beads were detected on western blots. Band intensities were quantified in LICOR, normalized to talin binding in the absence of kinldin-3, and presented as a curve. Insert shows a representative western blot of 3 independent experiments. Increasing amounts of β3 tail-bound kindlin-3 failed to promote β3–talin interaction. Data represent means ± SEM of three experiments. (**F**) Same as (**E**) but purified THD was used instead of talin. Increasing amount of β3 tail-bound kindlin-3 failed to promote β3–THD interaction. Blots were performed with anti-talin (8d4) for talin, anti-flag and anti-His6 for kindlin-3 and THD. β3 tail loading was visualized by Coomassie stain. (asterisks in E and F denote statistically significant differences compared to talin or THD binding in the absence of kindlin-3, P<0.05).

Since talin recruitment to membranes may be necessary but not sufficient for talin recruitment to αIIbβ3 (Figure 1), we employed bimolecular fluorescence complementation [48], [49] to evaluate whether kindlins promote talin recruitment to the integrin [11]. The principle of BiFC for studies of talin recruitment is illustrated in Figure 4A. For these experiments, the C-terminal half of Venus fluorescent protein was fused to the C-terminus of αIIb and this fusion protein was stably expressed in CHO cells with β3 (αIIb-VCβ3 CHO cells). The N-terminal half of Venus was fused to the N-terminus of talin and VN-talin was expressed conditionally in αIIb-VCβ3 CHO cells in response to doxycycline. When VN-talin interacts with αIIb-VCβ3, the split Venus moieties should come together, re-fold and generate fluorophore detectable by flow cytometry or microscopy. To control for fluorescence due to “non-specific” Venus self-association independent of talin interaction with β3 [50], parallel experiments were carried out with αIIb-VCβ3Δ724 CHO cells, in which the β3 tail had been truncated to delete residues necessary for initial interactions with talin and kindlins [11]. To control for potential differences in αIIbβ3 expressions, BiFC signals were first normalized to αIIbβ3 expression levels, as measured with an anti-αIIbβ3 antibody (D57), and then compared for each experimental condition.

Before treatment with doxycycline, αIIb-VCβ3 CHO cells exhibited a basal level of Venus fluorescence, likely due to leaky expression of VN-talin (Figure 4B). After induction of VN-talin expression with doxycycline, BiFC increased significantly in the αIIb-VCβ3 CHO cells (P<0.01) but not in the αIIb-VCβ3Δ724 CHO cells (Figure 4B). Furthermore, transient over-expression of kindlin-2 in the αIIb-VCβ3 CHO cells failed to increase BiFC further; rather, a small decrease was observed. A small but statistically significant decrease in BiFC was also obtained when kindlin-3 was over-expressed (P<0.05) (Figure 4B). Since kindlin over-expression did not affect VN-talin expression (Figure 4C), these results suggest that kindlins do not promote the interaction of talin with αIIbβ3 in CHO cells, despite the fact that kindlin-2 promotes talin-dependent αIIbβ3 activation (Figure 2) [21]. This conclusion is tempered by the possibility that endogenous kindlin-2 in the cells may be sufficient to promote talin recruitment to β3. To examine this possibility, αIIb-VCβ3 CHO cells were transduced with a *kindlin-2* (or control) shRNA, and VN-talin expression was induced with doxycycline. Although knockdown of kindlin-2 was efficient (Figure 4C) and caused a decrease in PAC-1 binding (Figure 4D) qualitatively similar to our previous result obtained in a different cell clone (Figure 2A), it had no effect on the interaction between VN-talin and αIIb-VCβ3 (Figure 4B), indicating that talin recruitment to αIIbβ3 in CHO cells does not require the presence of kindlin. Although over-expressed kindlins inhibited talin-integrin interactions, endogenous kindlin might not be sufficient to block talin binding. This could account for the failure of kindlin knockdown to increase talin-integrin BiFC.

Since BiFC experiments require Venus fusion proteins, it could be argued that the above results do not reflect the influence of kindlin-2 on the behavior of native talin or αIIbβ3. Therefore, these observations were extended to purified, recombinant proteins. We used recombinant kindlin-3 (Figure S2), the hematopoietic isoform that is physiologically relevant to integrin αIIbβ3 regulation and has 53% identity and 72% similarity to kindlin-2, for the *in vitro* experiments. When talin or THD, the integrin fragment of talin that activates integrins, was incubated with beads coated with recombinant β3 cytoplasmic tail, it bound to the beads as expected [51]. Moreover, increasing amounts of β3 tail-bound kindlin-3 failed to promote either talin or THD binding to the β3 tail (Figure 4E,F). In fact, kindlin appears to inhibit the binding of talin but not THD (Figure 4E,F), to β3 tails, suggesting that the inhibitory effects might be a result of steric hindrance. This result is consistent with the inhibitory effects of kindlins on talin recruitment to αIIbβ3 we observed in the BiFC studies (Figure 4B). Thus, we conclude that kindlins do not promote talin interaction with αIIbβ3, either in CHO cells or in a purified system.

**Figure 5:**
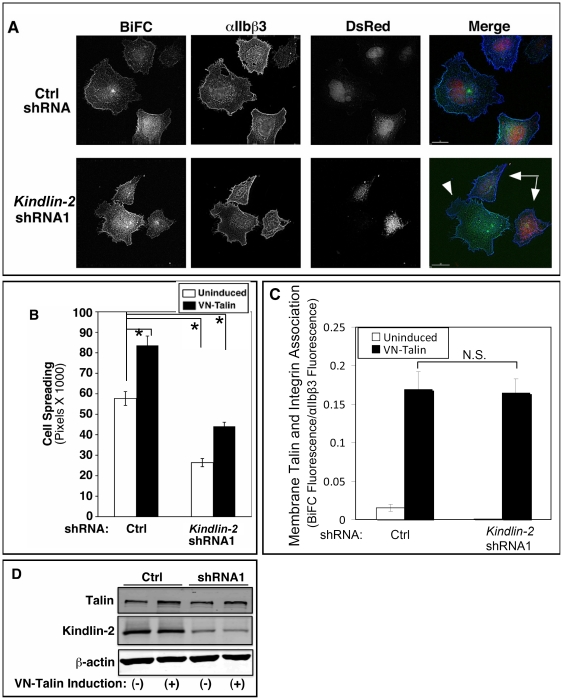
Subcellular localization of BiFC signals. αIIb-VCβ3 CHO cells were transduced with *kindlin*-2 (or control) shRNA lentiviruses also encoding DsRed, and VN-talin was induced by doxycycline. (**A**) Cells were incubated on fibrinogen-coated plates (100 µg/ml coating concentration) for 45 min, fixed, stained with antibody D57 for αIIbβ3, and examined by deconvolution microscopy (BiFC: Green, αIIbβ3: blue, Transduced: red). The arrows point to transduced cells, and the arrowhead to a non-transduced cell. (**B**) Spreading of transduced cells was examined and data were expressed as mean cell surface areas measured in total pixels as described in Experimental Procedures. Asterisk denotes statistically significant difference against respective control cells, P<0.01 (**C**) BiFC and αIIbβ3 fluorescence co-localization in transduced cells was evaluated by deconvolution microscopy as described in Experimental Procedures. Data represent 30–60 cells analyzed for each treatment. (**D**) Western blots were performed to monitor expression of talin and kindlin-2 in cell lysates. The cell lysates were from both uninfected and virus transduced cells whereas only virus transduced cells were analyzed in (A), (B) and (C).

To visualize the cellular location of the BiFC signals, αIIb-VCβ3 CHO cells were plated on fibrinogen for 45 min to allow cell spreading, and cells were examined by fluorescence microscopy. As expected, induction of VN-talin expression increased cell spreading, whereas kindlin-2 knockdown reduced cell spreading (P<0.01) (Figure 5A,B). Despite this, kindlin-2 knockdown had no effect on the intensity of BiFC signals co-localizing with antibody-stained αIIbβ3 at the plasma membrane, consistent with our subcellular fractionation results (Figure 5A,C). Although cell spreading is significantly reduced in kindlin-2 knock-down cells, cell spreading increased in these cells in response to induction of VN-talin expression. This could be a result of increased αIIb-VCβ3 and VN-talin interaction due to basal VN-VC self-complementation. Alternatively, we speculate that kindlin-dependency of cell spreading (outside-in-signaling) and integrin activation (inside-out signaling) may differ.

**Figure 6:**
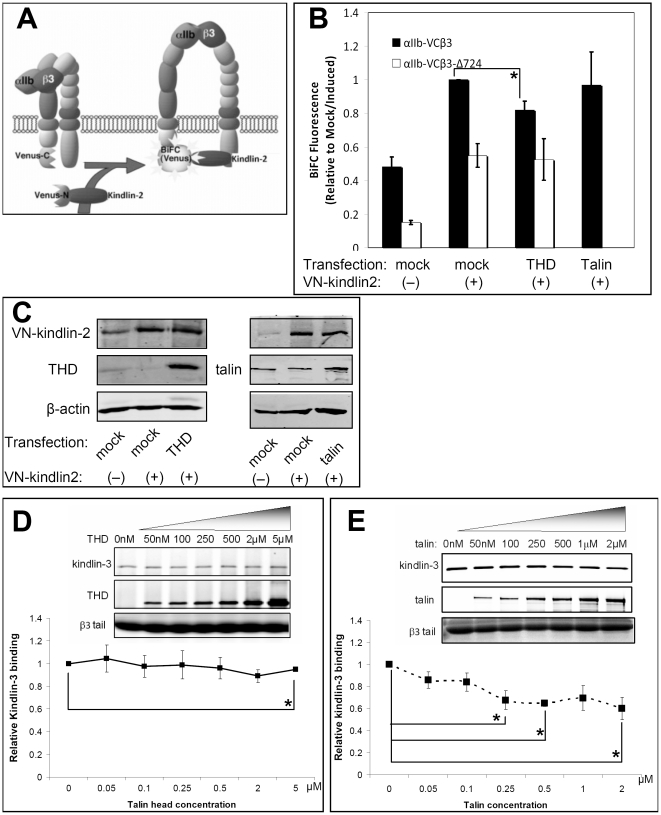
Effect of talin or THD on interaction between kindlins and αIIbβ3. (**A**) Schematic illustration of BiFC in CHO cells depicting αIIb-VC, VN-kindlin-2 and β3. When VN-kindlin interacts with αIIb-VCβ3 through the β3 tail, VN and VC should reconstitute Venus, resulting in BiFC. (**B**) Over-expression of THD or talin does not promote kindlin-2 interaction with αIIbβ3. CHO cells expressing αIIb-VCβ3 or αIIb-VCβ3Δ724 was co-transfected with Tac as a transfection marker and an expression vector for THD, talin or empty vector (Mock), as indicated. After induction of VN-kindlin-2 expression with doxycycline, BiFC was quantified by flow cytometry. BiFC fluorescence was normalized to αIIbβ3 expression and expressed as fold-increase relative to doxycycline-treated, Mock-transfected cells. Data represent means ± SEM of three experiments (asterisk denotes statistically significant difference against mock/induced, P<0.05). (**C**) Western blots were performed to monitor expression of VN-kindlin-2, talin and THD in cell lysates. (**D**) Purified kindlin-3 with or without addition of THD was incubated with the recombinant β3 cytoplasmic tail conjugated to neutravidin beads. After washing, proteins bound to the beads were detected on western blots. Band intensities were quantified in LICOR, normalized to kindlin-3 binding in the absence of THD, and presented as a curve. Insert shows a representative western blot of 3 independent experiments. Increasing amount of β3 tail bound THD failed to promote β3–kindlin-3 interaction. Data represent means ± SEM of three experiments. (**E**) Similar to (D) but talin was used instead of THD. Increasing amounts of β3 tail-bound talin failed to promote β3–kindlin-3 interaction. (asterisks in D and E denotes statistically significant differences compared to kindlin binding in the absence of talin or THD, P<0.10).

Although the PH domain and positively charged motifs in the F1 domain of kindlins facilitate targeting to membrane phosphoinositides [44], [52], [53], mechanisms regulating the interaction of kindlins with integrin cytoplasmic tails remain to be fully characterized. We modified the BiFC method to ask whether the interaction of kindlins with the β3 cytoplasmic tail is influenced by talin binding to β3 (Figure 6A). VN-kindlin-2 was expressed conditionally in αIIb-VCβ3 CHO cells and BiFC was studied by flow cytometry. Preliminary experiments established that VN-kindlin-2 was functional in that it promoted talin-dependent PAC-1 binding to doxycycline-treated αIIb-VCβ3 CHO cells (not shown). Induction of VN-kindlin-2 expression in these cells caused a significant increase in BiFC fluorescence to a much greater extent than in αIIb-VCβ3Δ724 CHO cells, showing that VN-kindlin-2 and αIIb-VCβ3 interaction requires the β3 cytoplasmic domain (Figure 6B). However, over-expression of either THD or full-length talin failed to increase the interaction between kindlin-2 and αIIb-VCβ3 (Figure 6B,C). On the contrary, over-expression of THD inhibited BiFC between VN-kindlin-2 and αIIb-VCβ3 (Figure 6B). Studies with native, recombinant proteins *in vitro* demonstrated that increasing concentrations of THD had no effect on the binding of kindlin-3 to the β3 cytoplasmic tail (Figure 6D). The reason for this discrepancy, we speculate, is that in cells integrin activation by THD results in other signaling factors or signaling complexes being recruited to integrin tails, which might then affect kindlin-integrin interaction. Consistent with our previous observation that recombinant kindlin-3 competes with purified talin in binding to the β3 tail (Figure 4F), recombinant talin also inhibited kindlin-3 and β3 tail interaction (Figure 6E). However, over-expression of talin in CHO cells did not inhibit BiFC between VN-kindlin2 and αIIb-VCβ3 (Figure 6B), possibly because only a small fraction of over-expressed talin was targeted to the plasma membrane (Figure 3A,B,S3). Overall, we conclude that talin does not promote kindlin interaction with integrin β3.

In conclusion, the present studies in CHO cells and in purified systems suggest that kindlins and talin do not promote each other's bulk interactions with integrin β3. Whereas it is not currently feasible to conduct similar studies in platelets, we speculate that kindlins might co-activate αIIbβ3 in platelets by participating in events other than initial talin recruitment to integrin αIIbβ3. THD is sufficient to activate αIIbβ3 in model membranes in purified systems [17] and can activate the integrins in cells when the integrins' kindlin binding site is mutated or endogenous *kindlin* is silenced. Yet kindlins further promote talin-dependent αIIbβ3 activation in cells [18], [21], [22]. Consequently, a fundamental question remains as to how the kindlins function as αIIbβ3 co-activators if they do not promote talin targeting to membranes or to the integrin itself. A related question is why the integrin co-activating functions of kindlin-2 and kindlin-3 are cell-type and integrin-specific [21], [22], [54]. Based on the present studies, we suggest that talin and kindlin synergize in integrin activation not by altering the interaction of each other with the integrins. Thus, kindlins may co-activate αIIbβ3 by modifying events that take place after initial talin recruitment (Figure 1). Such events might include 1) post-translational or structural changes in talin or αIIbβ3that promote talin interactions with membrane-proximal residues in the β3 tail or with membrane phospholipids; 2) displacement of a negative regulator of integrin signaling that competes with talin for critical interactions with αIIbβ3; or 3) interaction of talin and kindlins together with as yet unidentified factor(s) to promote integrin activation [2], [20], [42].

## Supporting Information

Figure S1 ***kindlin-2*** **shRNA can achieve efficient kindlin-2 knockdown in CHO cells.** Integrin expressing CHO cells were transduced with lentivirus encoding control or *kindlin*-2 shRNAs as described in Experimental Procedures. 96 hours later the cells were analyzed by western blotting to determine the efficiency of kindlin-2 knockdown (**A**) and by FACS to determine the percentage of lentivirus transduced cells (GFP positive) (**B**). Numbers in (A) indicates kindlin-2 band intensity. *Kindlin-2* shRNA1 achieved 83% kindlin-2 depletion with 92% infection rate, indicating that shRNA1 transduced cells on average lost 90% of kindlin-2 expression. ShRNA2 resulted in 65% depletion with 65% infection rate, indicating that shRNA2 transduced cells lost virtually all of their kindlin-2 expression. The lane of shRNA2 was excised from the same blot image and was juxtaposed to the other two lanes for clarity. Monoclonal anti-kindlin-2 from Dr. Wu (University of Pittsburg) was used for kindlin-2 detection.
(TIF)

Figure S2 **Characterization of recombinant kindlin3.** Biophysical and biochemical assays indicated that recombinant kindlin-3 was monomeric and folded. (A) Size exclusion chromatography of purified kindlin-3 compared to molecular standards. (B) Coomassie Brilliant Blue staining of purified kindlin-3, showing a major band consistent with the theoretical molecular size of the kindlin-3 construct, 76.9 kDa. (C) Plot of the Stokes radius of the standard against (−Log((Ve-Vo)/(Vc-Vo)))(1/2), where Ve represents retention volume, Vo represents void volume, and Vc represents column volume. Stokes radii used for the standard proteins were: thyroglobulin 8.5 nm, apoferritin 6.1 nm, β-Amylase 5.4 nm, albumin 3.55 nm, and carbonic anhydrase 2.01 nm. The calculated Stokes radius for kindlin-3 is ∼3.8 nm, and the kindlin-3 chromatogram is consistent with a molecular weight of 91.6 kDa, assuming kindlin-3 is a globular protein similar to the standards. (D) Differential scanning calorimetry showing two peaks indicating that recombinant kindlin-3 is folded. It is likely that the first peak is the melting of the protein tertiary structure and the second peak the melting of a stable secondary structure or structural domain.
(TIF)

Figure S3 **Representative western blots of the subcellular fractionation experiments.** WCL, nuclei and unbroken cells, cytosolic fraction, crude membrane fraction and plasma membrane fraction were resolved by SDS-PAGE, transferred to nitrocellulose membrane, and blotted by anti-kindlin-2, anti-talin, anti-HA, anti-αIIb. Calnexin as an endoplasmic reticulum marker and RhoGDI as a cytosolic marker and loading control were also blotted to assess the purity of the plasma membrane preparation.
(TIF)
