# Supplementary figures and images for: Kindlins, Integrin Activation and the Regulation of Talin Recruitment to αIIbβ3
Source: PLoS One. 2012 Mar 23;7(3):e34056. doi: 10.1371/journal.pone.0034056 (PMC3311585; doi:10.1371/journal.pone.0034056)

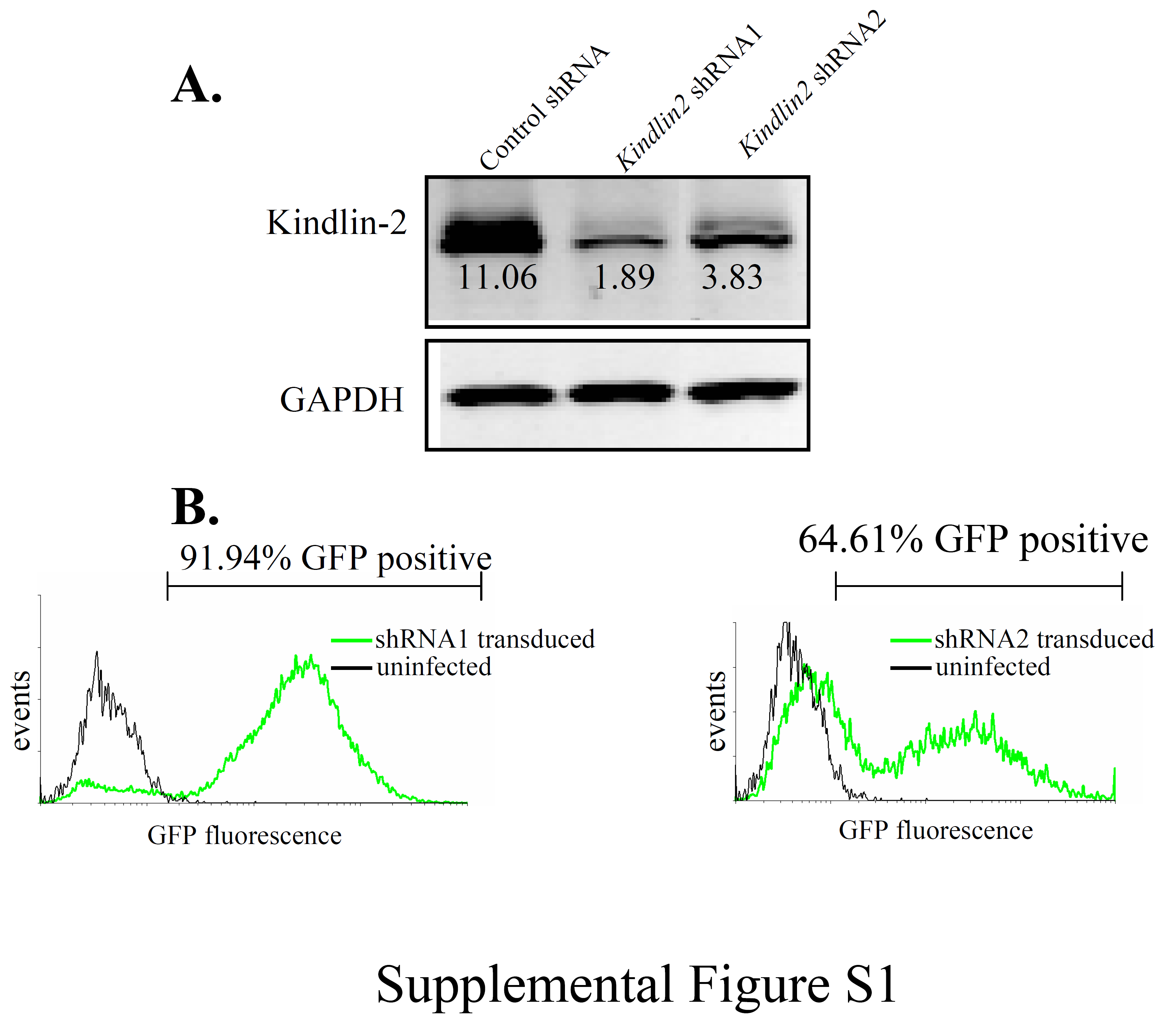

Supplement: Figure S1 — kindlin-2 shRNA can achieve efficient kindlin-2 knockdown in CHO cells. Integrin expressing CHO cells were transduced with lentivirus encoding control or kindlin-2 shRNAs as described in Experimental Procedures. 96 hours later the cells were analyzed by western blotting to determine the efficiency of kindlin-2 knockdown (A) and by FACS to determine the percentage of lentivirus transduced cells (GFP positive) (B). Numbers in (A) indicates kindlin-2 band intensity. Kindlin-2 shRNA1 achieved 83% kindlin-2 depletion with 92% infection rate, indicating that shRNA1 transduced cells on average lost 90% of kindlin-2 expression. ShRNA2 resulted in 65% depletion with 65% infection rate, indicating that shRNA2 transduced cells lost virtually all of their kindlin-2 expression. The lane of shRNA2 was excised from the same blot image and was juxtaposed to the other two lanes for clarity. Monoclonal anti-kindlin-2 from Dr. Wu (University of Pittsburg) was used for kindlin-2 detection. (TIF) [file pone.0034056.s001.tif]

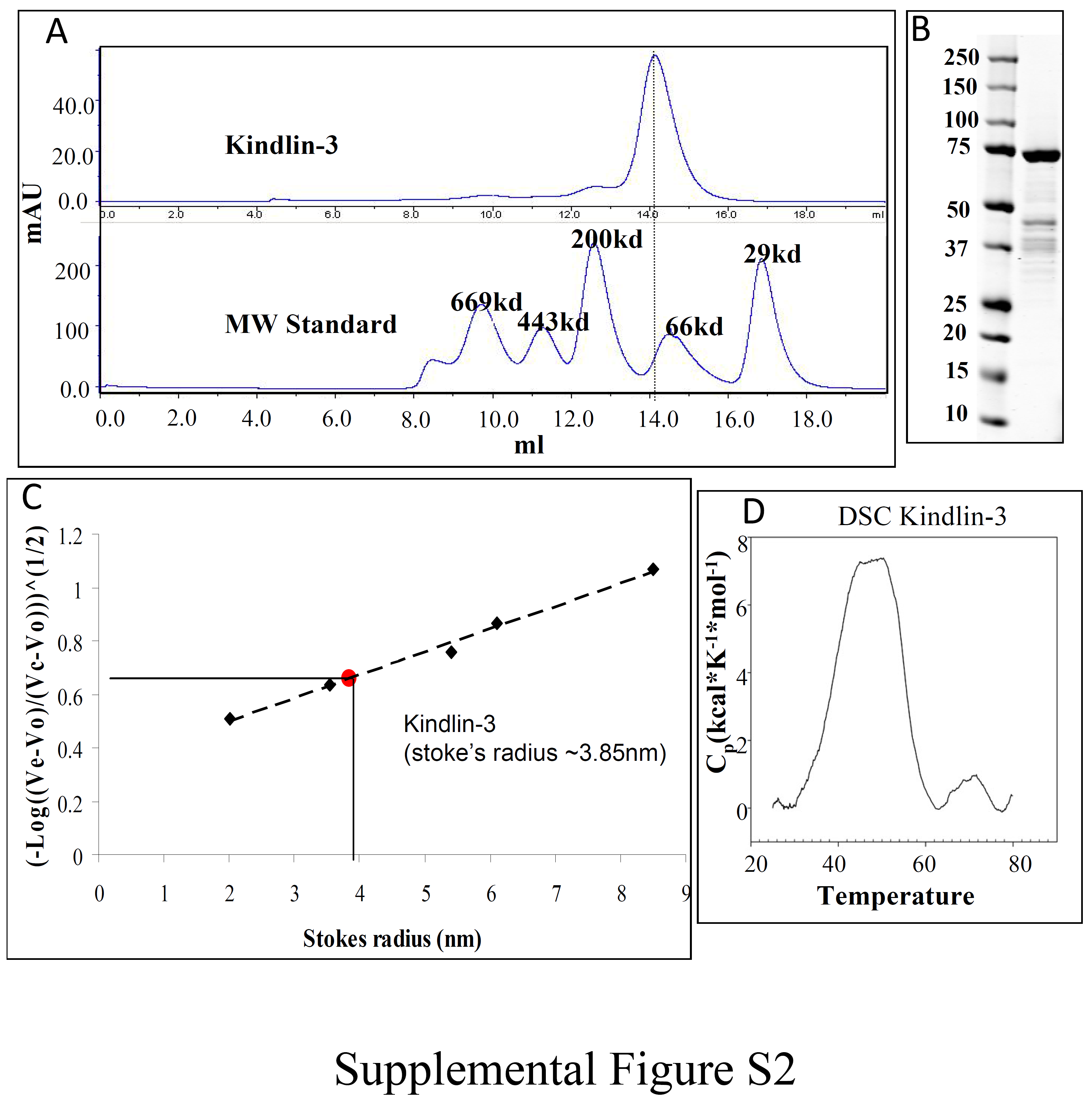

Supplement: Figure S2 — Characterization of recombinant kindlin3. Biophysical and biochemical assays indicated that recombinant kindlin-3 was monomeric and folded. (A) Size exclusion chromatography of purified kindlin-3 compared to molecular standards. (B) Coomassie Brilliant Blue staining of purified kindlin-3, showing a major band consistent with the theoretical molecular size of the kindlin-3 construct, 76.9 kDa. (C) Plot of the Stokes radius of the standard against (−Log((Ve-Vo)/(Vc-Vo)))(1/2), where Ve represents retention volume, Vo represents void volume, and Vc represents column volume. Stokes radii used for the standard proteins were: thyroglobulin 8.5 nm, apoferritin 6.1 nm, β-Amylase 5.4 nm, albumin 3.55 nm, and carbonic anhydrase 2.01 nm. The calculated Stokes radius for kindlin-3 is ∼3.8 nm, and the kindlin-3 chromatogram is consistent with a molecular weight of 91.6 kDa, assuming kindlin-3 is a globular protein similar to the standards. (D) Differential scanning calorimetry showing two peaks indicating that recombinant kindlin-3 is folded. It is likely that the first peak is the melting of the protein tertiary structure and the second peak the melting of a stable secondary structure or structural domain. (TIF) [file pone.0034056.s002.tif]

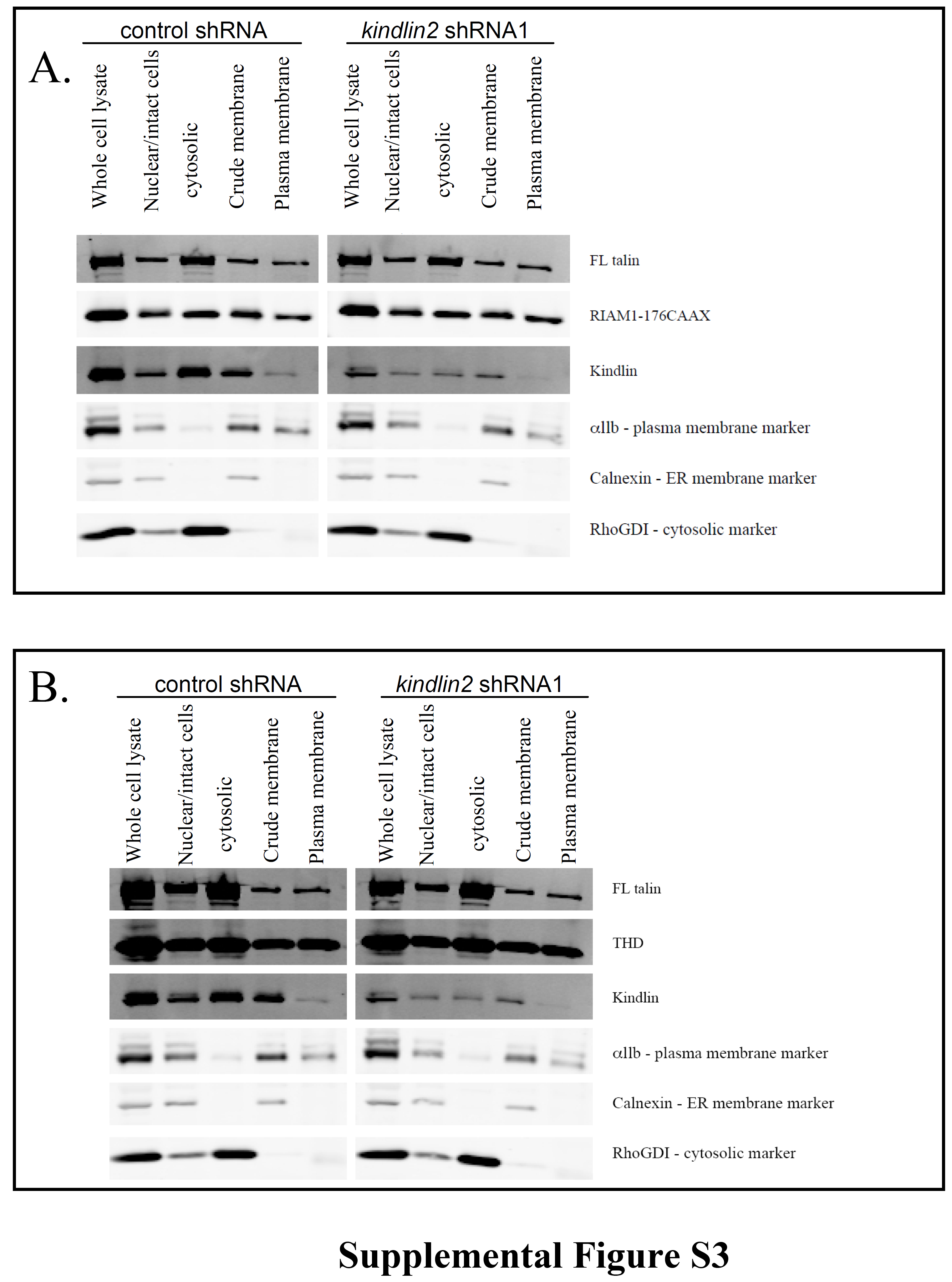

Supplement: Figure S3 — Representative western blots of the subcellular fractionation experiments. WCL, nuclei and unbroken cells, cytosolic fraction, crude membrane fraction and plasma membrane fraction were resolved by SDS-PAGE, transferred to nitrocellulose membrane, and blotted by anti-kindlin-2, anti-talin, anti-HA, anti-αIIb. Calnexin as an endoplasmic reticulum marker and RhoGDI as a cytosolic marker and loading control were also blotted to assess the purity of the plasma membrane preparation. (TIF) [file pone.0034056.s003.tif]
